# Knockdown of Matrix Metallopeptidase 9 Inhibits Metastasis of Oral Squamous Cell Carcinoma Cells in a Zebrafish Xenograft Model

**DOI:** 10.1155/2020/4350783

**Published:** 2020-04-15

**Authors:** Jinlin Wen, Panpan Yin, Linwei Li, Guihua Kang, Guozhu Ning, Yu Cao, Feng Gao, Ying Su, Yanlin Wu, Xinyan Zhang

**Affiliations:** ^1^Beijing Institute of Dental Research, Beijing Stomatological Hospital & School of Stomatology, Capital Medical University, Beijing, China; ^2^State Key Laboratory of Membrane Biology, Institute of Zoology, University of Chinese Academy of Sciences, Chinese Academy of Sciences, Beijing, China

## Abstract

Destruction of extracellular matrix (ECM) is one of the basic steps of tumor invasion and metastasis. Matrix metalloproteinase (MMP) 9, a kind of zinc-ion-dependent endopeptidase, can degrade almost all protein components in the ECM, destroy the histological barrier of tumor cell invasion, and play a key role in tumor invasion and metastasis. The role of MMP-9 in tumor invasion and metastasis has attracted increasing attention and is considered the main proteolytic enzyme in this process. Although the overexpression of MMP-9 was detected in Oral squamous cell carcinoma (OSCC) tissues, further basic studies *in vivo* and *in vitro* are needed to investigate the role of MMP-9 in OSCCs and provide scientific validation. In this research, we developed a novel OSCC zebrafish xenograft model to study the role of the MMP-9 gene in oral carcinogenesis. Firstly, the MMP-9/shRNA lentiviral clone and control virus were constructed and transfected into OSCC cells. Then, the decreasing expression of MMP-9 was verified by RT-PCR and immunocytochemistry. Cell proliferation was detected by MTT assay. Colony formation was evaluated by colony formation assay. Cell invasion was evaluated using transwell invasion assay *in vitro*. In addition, OSCC cells with MMP-9/shRNA knockdown and control vector were injected into zebrafish and an OSCC tumor model in zebrafish was established to evaluate invasion and metastasis *in vivo*. Knockdown of MMP-9 gene by shRNA could inhibit OSCC cell growth and clone formation and markedly suppress cell invasion *in vitro*. And the knockdown of the MMP-9 gene could also significantly decrease the metastatic distance and number of metastatic tumor cells or lesions *in vivo* and suppress the metastasis rate in xenografted zebrafish. Taken together, these evidences indicated that the knockdown of MMP-9 might suppress OSCC cell invasion and metastasis *in vivo* and *in vitro*. The MMP-9 gene may be a promising therapeutic target for OSCCs in the future.

## 1. Introduction

Oral squamous cell carcinoma (OSCC) is one of most common types of head and neck cancer and about two-thirds of deaths are in developing countries [1]. Despite multidisciplinary treatment, the survival rate of patients is still only approximate 50% [2]. Metastasis is one of the main causes of high mortality in OSCC patients. The OSCC cells tend to spread initially to regional lymph nodes in the neck before they spread to distant sites (lung or other organs) [3]. Therefore, inhibiting tumor metastasis is critical for improving clinical outcomes of OSCC patients.

Extracellular matrix (ECM), a highly dynamic noncellular component in tissue, can provide both biochemical and essential structural support for cellular components [4]. For cancers to commence invasion and metastasis, they must break the ECM barrier of the basement membrane [5]. Matrix metalloproteinases (MMPs), the family of calcium-dependent and zinc-containing endopeptidases, can break the ECM barrier in physiological processes and in cancers [6]. Among all MMPs, MMP-9 is considered the especially compelling target and main proteolytic enzyme in this process, which regulates the pathological remodeling, degrades almost all protein components in the ECM, destroys the histological barrier of tumor cell invasion, and plays a central role in cancer invasion and metastasis [7]. In the previous studies, overexpression of MMP-9 has been reported to be associated with higher grade, metastasis, and angiogenesis in a large number of human cancers [8, 9]. In particular, decreasing expression of MMP-9 could inhibit the invasive and metastatic ability of some cancers, such as pancreatic adenocarcinoma [10], lung cancer [11], thyroid cancer [12], and ovarian cancer [13]. But MMP-9 could also maintain the epithelial-mucosal integrity by activating the EGFR-Sp1 signaling pathway, thereby playing a suppressor role in colitis-associated cancer [14]. The overexpression of MMP-9 in human OSCC tissues may contribute to cancer metastasis, suggesting that MMP-9 may serve as a prognostic biomarker of OSCC [15, 16]. In addition, the elevated MMP-9 level in saliva may be a useful diagnostic factor for the early detection of OSCC [17, 18]. Although the high expression of MMP-9 was considered to be associated with the invasion of OSCCs in clinical studies, further basic studies *in vivo* and *in vitro* are necessary to confirm the role of MMP-9 in OSCCs and provide scientific validation.

The zebrafish, first described by George Streisinger, emerged as an animal model of developmental genetics in the 1960s. Since then, zebrafish has been widely used in biomedical studies and has become an increasingly important cancer model [19]. Compared with other animal models, zebrafish provides a promising opportunity to monitor the dissemination and metastasis of single tumor cell owing to these advantages including large number of transparent eggs, rapid external development, immune-privileged feature, and less raised cost [20, 21]. Recently, a series of zebrafish cancer models had been developed including uveal melanoma model [22], conjunctival melanoma model [23], glioma model [24], breast cancer model [25], malignant melanoma model [26], liposarcoma cancer model [27], and leukemia model [28]. Furthermore, zebrafish can absorb small molecular compounds from water and play an important role in efficiently and easily screening a large number of anticancer compounds [29]. In this study, we established a novel human OSCC model in zebrafish body to observe tumor progression in a real-time manner and confirm the possible function of MMP-9 *in vivo*. Our observations revealed that knockdown of MMP-9 might play the inhibitor role in the growth, invasion, and metastasis of OSCC cells *in vivo* and *in vitro*. Moreover, the MMP-9 gene may be a promising therapeutic target for OSCCs in the future.

## 2. Materials and Methods

### 2.1. Cell Line Culture

The OSCC cell lines CAL-27 and SCC-15 were obtained from the Wuhan University and American Type Culture Collection (ATCC, Manassas, VA), respectively. The OSCC cells were routinely cultured in DMEM : F12 or DMEM high-glucose medium (Invitrogen Life Science, Carlsbad, CA) containing 10% fetal bovine serum (FBS), 100 U/ml penicillin, and 100 *μ*g/ml streptomycin with 5% CO_2_ at 37°C. All *in vitro* experiments were done in triplicate to ensure reliability.

### 2.2. Cell Line Transfection

The MMP-9/shRNA lentiviral clone and control shRNA vector were obtained from GeneCopoeia Inc. (Rockville, MD) (listed in Supplementary Figure 1). After confirming the knockdown of MMP-9, the most efficient shRNA sequence was selected for subsequent experiments. Then, the lentiviral knockdown plasmid using HIV, psi-LVRU6MP/MMP-9/shRNA, and Lenti-Pac HIV Expression Packaging Kit was cotransfected into 293Ta cells to package lentiviral particles and produce recombinant lentiviral particles. SCC-15 cells (1.5 × 10^5^/ml) and CAL-27 cells (6 × 10^4^/ml) were seeded into 6-well plates one day before transfection. Then, the OSCC cell lines were transfected with lentiviruses containing MMP-9/shRNA. The lentivirus transfer vector expressing mCherry protein served as the control. To obtain stable lentiviral transduction of OSCC cell lines, we screened the OSCC cells with puromycin (Sigma-Aldrich, Saint Louis, MO). There was no significant change in the viability and growth of OSCC cells after labeling.

### 2.3. Real-Time PCR (RT-PCR) Analysis

The effect of decreasing MMP-9 expression at the RNA level was verified by RT-PCR. Trizol reagent (ComWin Biotech Co., Ltd., CHN) was used to isolate the total RNA from transfected OSCC cells. Super RT cDNA Synthesis kit (ComWin Biotech) was used to get single-stranded cDNA. Then, ULtraSYBR Mixture kit (Low ROX) (ComWin Biotech) was used for quantitative PCR following the manufacturer's instructions. The PCR primer sets for human MMP-9 were provided by GeneCopoeia Inc. And GAPDH (forward, 5′-catgggtgtgaaccatgagaagtat-3′; reverse, 5′-gactgtggtcatgagtccttcca-3′) were provided from Sangon Biotech Co., Ltd. (CHN). The *ΔΔ*Ct method was used for quantification, and data were normalized to GAPDH (served as the endogenous control).

### 2.4. Immunocytochemistry

Reduced expression of MMP-9 at the protein level was detected by immunocytochemistry. The control vector cells and shRNA cells (2 × 10^4^/ml) were, respectively, seeded in 24-well plates and cultured at 37°C with 5% CO_2_. Then, cells were fixed with 10% neutral buffer formalin fixative for 10 mins and incubated with Triton X-100 for 10 mins. And cells were blocked in PBS mixture containing 10% goat serum for 1 h. Then, cells were incubated with mouse polyclonal anti-MMP-9 antibody (Proteintech Group, Inc., Rosemont, IL, 1 : 1000) overnight at 4°C. After cells were wasted with PBS, they were treated with hydrogen peroxide for 10 mins and stained with diaminobenzidine (DAB) kit. The pictures were taken under an inverted microscope.

### 2.5. MTT Assay

The growth of OSCC cells were assessed by MTT assay. CAL-27/LVRU6MP (served as the control) and CAL-27/MMP-9/shRNA cells (3 × 10^4^/ml) and SCC-15/LVRU6MP (served as the control) and SCC-15/MMP-9/shRNA cells (1 × 10^4^/ml) were, respectively, seeded in 96-well plates and cultured for 24 h, 48 h, and 72 h in an incubator with 5% CO_2_. After adding 20 *μ*l MTT solution (5 mg/ml) in 96-well plates, the cells were incubated for 4 h. To dissolve reactant, 200 *μ*l dimethyl sulfoxide (DMSO, Sigma-Aldrich Corp) was added to each well. Then, these plates were read at a wavelength of 490 nm using a microplate reader (Sunnyvale, CA), and the optical density (OD) value was measured.

### 2.6. Colony Formation Assay

The colony formation ability of OSCC cells was evaluated by colony formation assay. The 100 *μ*l CAL-27/LVRU6MP and CAL-27/MMP-9/shRNA cell (1 × 10^4^/ml) and SCC-15/LVRU6MP and SCC-15/MMP-9/shRNA cell (5 × 10^3^/ml) suspensions were, respectively, prepared and seeded in 60 mm culture dishes. After 10 days of incubation, the cells were fixed with 10% neutral buffer formalin fixative and stained with crystal violet staining solution (Beyotime, CHN). To count the numbers, colonies were photographed.

### 2.7. Transwell Invasive Assay

The invasive ability of OSCC cells was assessed by transwell invasive assay. Matrigel (BD Biosciences, Franklin Lakes, NJ) was added into the upper chamber (8 *μ*m pore size; Corning, Corning, NY) of 24-well plates. The CAL-27/LVRU6MP and CAL-27/MMP-9/shRNA cells (1 × 10^6^/ml) and SCC-15/LVRU6MP and SCC-15/MMP-9/shRNA cells (5 × 10^6^/ml) were, respectively, suspended in the serum-free medium containing 2% bovine serum albumin (BSA, VWR, Radnor, PA) and transferred to the upper chamber above Matrigel. Then, the routine medium containing 10% FBS was added to the lower chamber. CAL-27/LVRU6MP and CAL-27/MMP-9/shRNA cells were cultured for 48 h, and SCC-15/LVRU6MP and SCC-15/MMP-9/shRNA cells were cultured for 72 h, respectively. The remaining cells that had not passed through Matrigel in the upper chamber were removed by cotton balls. Then, cells were fixed with 10% neutral buffer formalin fixative and stained with crystal violet staining solution. The pictures were taken under an inverted microscope for counting the numbers.

### 2.8. Zebrafish Maintenance

Wild-type (WT) Tuebingen (TU) strain of zebrafish was provided by State Key Laboratory of Membrane Biology, Institute of Zoology, University of Chinese Academy of Sciences, Chinese Academy of Sciences, and raised at 29°C under standard laboratory conditions. The embryos were collected and cultured in egg water containing 0.003% phenylthiourea (PTU, Sigma-Aldrich Corp). All *in vivo* experiments with zebrafish were repeated three times.

### 2.9. The Establishment of a Zebrafish OSCC Model

Before implantation, the CAL-27/LVRU6MP and CAL-27/MMP-9/shRNA cells and SCC-15/LVRU6MP and SCC-15/MMP-9/shRNA cells were, respectively, suspended in serum-free medium at a density of 4 × 10^7^/ml. At 48 hours postfertilization (hpf), dechorionated embryos were anesthetized with 0.4% tricaine (Sigma-Aldrich Corp) and placed in the petri dish covered with 1% low melting point agarose (Sigma-Aldrich Corp) in a certain order. Then, approximately 15 nl cell suspension (500-600 cells) was injected into the top of the yolk sac using glass capillary needles (approximately an opening size of a single-cell diameter) by micromanipulator (MPPI-3, Applied Scientific Instrumentation, Eugene, OR) (see Table 1 for details). These injected embryos were immediately transferred into 12-well plates (10 individuals per well) containing PTU egg water and maintained at 29°C. After half an hour of implantation, we discarded the embryos that have tumor cells mistakenly injected into the vascular system.

### 2.10. Microscopy and Image Analysis

To investigate the role of the MMP-9 gene in OSCC cells, these injected zebrafish were anesthetized and photographed daily in 3% methylcellulose using fluorescence stereoscopy (M205FA, Leica Microsystems, DE) from 0 to 6 days postinjection (dpi) (magnification, ×32, ×63). In brief, a red fluorescent focus indicated a metastatic tumor cell or lesion. The tumor metastatic distance was defined as the distance from the previous implantation location to the metastatic site [22]. Image J 1.0 (National Institutes of Health, Bethesda, MD) was used to measure the metastatic distance and count the number of metastatic cells or lesions. Additionally, the rate of metastasis was evaluated. It was calculated as follows: the metastasis rate (%) = (the number of zebrafish with tumor metastasis / the initial number of xenografted zebrafish) × 100%.

### 2.11. Statistical Analysis

Data were expressed as mean ± standard deviations (SD). Statistical analysis was performed by the Student *t*-test for paired comparisons and chi-square test for sample rates using SPSS Statistics 25.0 (IBM, Armonk, NY). *P* < 0.05 was deemed statistically significant.

## 3. Results

### 3.1. Establishment of a Knockdown Model of MMP-9 in OSCC Cells

**Figure 1:**
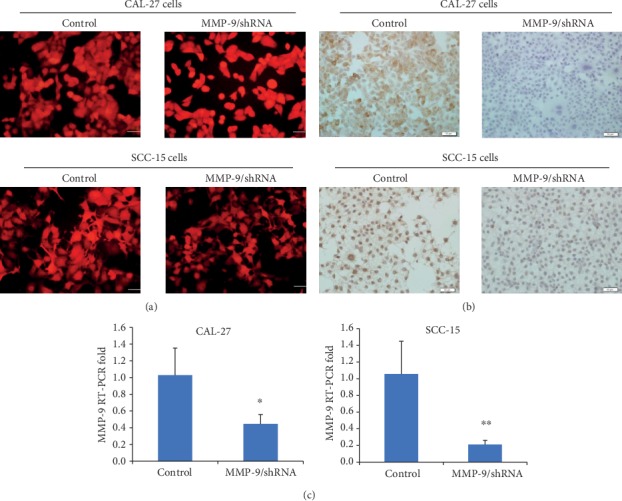
MMP-9 gene knockdown in OSCC cells. (a) The red fluorescence indicated the transfected cells (magnification, ×400). (b) Compared with the control, MMP-9 expression at the protein level was reduced in MMP-9/shRNA groups by immunocytochemistry analysis. Bar = 50 *μ*m. (c) The effect of decreasing MMP-9 expression at the RNA level was detected by RT-PCR. ^∗^*P* < 0.05 and ^∗∗^*P* < 0.01 as compared with the control. Bar = 200 mm.

To further study the role of the knockdown of MMP-9 in OSCC cells, we have constructed a mCherry-labeled OSCC cell knockdown model of MMP-9 (Figure 1(a)). The lentiviral vector information was provided in Supplemental [Supplementary-material supplementary-material-1]. Furthermore, RT-PCR and immunocytochemistry were performed to verify the decreasing expression of MMP-9. RT-PCR results showed that MMP-9 expression of the MMP-9/shRNA group was significantly reduced at the RNA level compared with the control (Figure 1(c)). Similarly, the level of MMP-9 protein in the MMP-9/shRNA group was also significantly lower than that in the shRNA control group (Figure 1(b)).

### 3.2. Knockdown of MMP-9 Suppresses Proliferation, Colony Formation, and Invasion of OSCC Cells In Vitro

**Figure 2:**
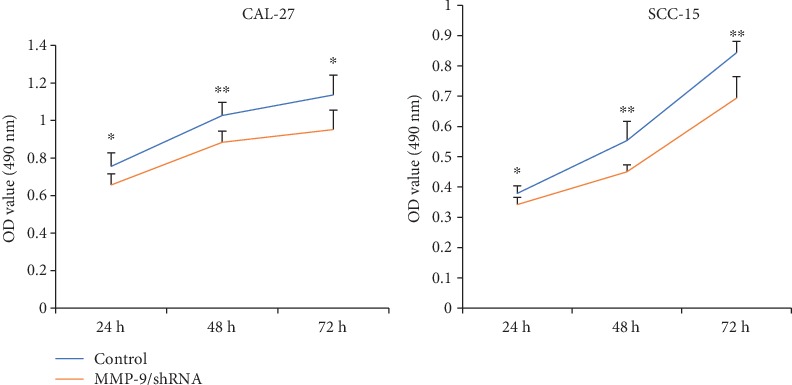
Knockdown of MMP-9 could slow down the growth of OSCC cells (CAL-27 and SCC-15 cells) in a time-dependent manner by MTT assay. ^∗^*P* < 0.05 and ^∗∗^*P* < 0.01 as compared with the control.

**Figure 3:**
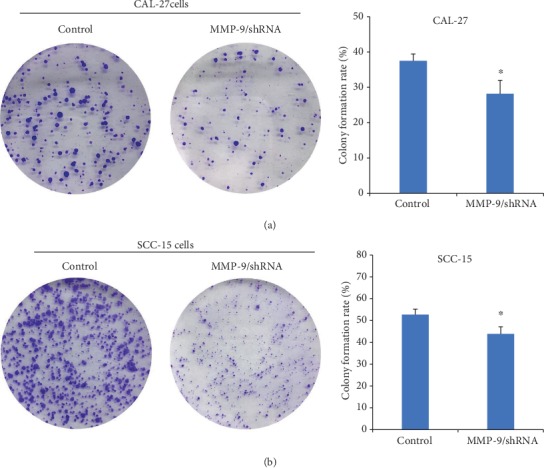
Knockdown of MMP-9 could inhibit the colony formation of OSCC cells (CAL-27 and SCC-15 cells) compared with the control. ^∗^*P* < 0.05 as compared with the control.

**Figure 4:**
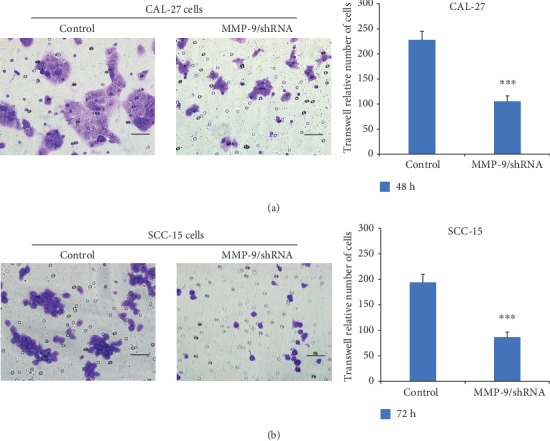
Knockdown of MMP-9 could decrease the invasive ability of OSCC cells (CAL-27 and SCC-15 cells) by transwell invasive assay (magnification, ×400). ^∗∗∗^*P* < 0.001 as compared with the control. Bar = 50 mm.

To further study the role of MMP-9 in OSCC cells *in vitro*, a series of experiments were performed. MTT assay results revealed that the knockdown of MMP-9 slowed down the proliferation of OSCC cells in a time-dependent manner (Figure 2). Additionally, colony formation assay results found that the knockdown of MMP-9 in OSCC cells inhibited the OSCC cell colony formation ability and reduced the size and number of colonies (Figure 3). Transwell invasive assay was used to investigate the invasive ability of OSCC cells. Matrigel acts as the basement membrane matrix, and cancer cells can only invade by destroying it. Compared with the control group, the knockdown of MMP-9 in OSCC cells significantly reduced the ability to invade (Figure 4). These data indicated that the knockdown of MMP-9 plays an inhibitor role in OSCC cell proliferation, colony formation, and invasion *in vitro*.

### 3.3. Knockdown of MMP-9 May Inhibit OSCC Cell Dissemination and Metastasis in a Zebrafish Xenograft Model

**Figure 5:**
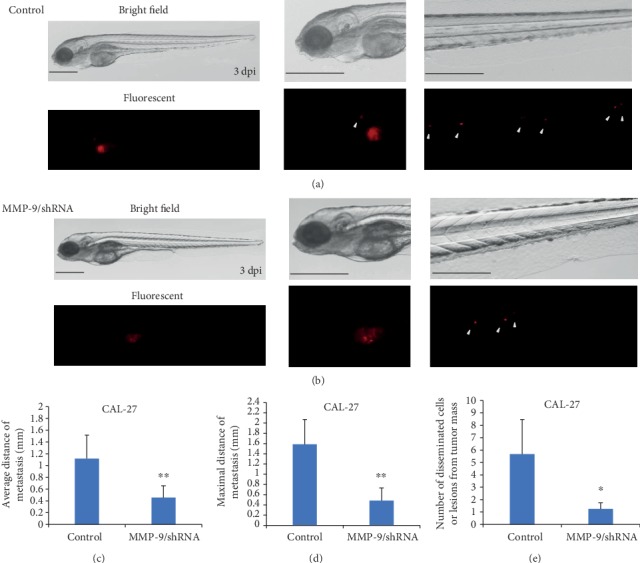
Knockdown of MMP-9 decreased the metastatic distance and number of CAL-27 cells in the zebrafish xenograft model. A red fluorescent cluster indicated a tumor mass, and the foci marked by white arrows indicated the metastatic tumor cells or lesions that have escaped from the yolk sac. (a, b) The bright-field and fluorescent pictures of whole-body, head, distal tail regions of zebrafish injected with CAL-27/LVRU6MP and CAL-27/MMP-9/shRNA cells at 3 dpi (*n* = 120; magnification, ×32, ×63). (c, e) Knockdown of MMP-9 reduced the invasive average distance, maximal distance, and number of disseminated cells or lesions at 3 dpi. ^∗^*P* < 0.05 and ^∗∗^*P* < 0.01 as compared with the control. Bar = 0.5 mm.

To further confirm the role of MMP-9 in OSCC cells *in vivo*, we established a novel human OSCC model in zebrafish by implanting tumor cells into the yolk sac of the embryo. The dissemination of OSCC cells out of the yolk sac is deemed as active metastasis and invasion because the yolk is considered an acellular and partly avascular environment that cannot support passive cell transport [22, 30]. At 3 dpi, some CAL-27/LVRU6MP cells escaping the yolk were observed in the pronephric region, trunk, and distal tail region (Figure 5(a)). But limited invasive CAL-27/MMP-9/shRNA cells were detected in the trunk region and showed less metastatic distance (Figure 5(b)). In order to quantify the metastatic ability of different OSCC cell lines in the zebrafish body, ImageJ 1.0 software was applied to picture analysis. Data analysis showed that the knockdown of MMP-9 significantly reduced the average metastatic distance, maximal distance, and number of metastatic tumor cells or lesions in the zebrafish body at 3 dpi (Figures 5(c)–5(e)).

**Figure 6:**
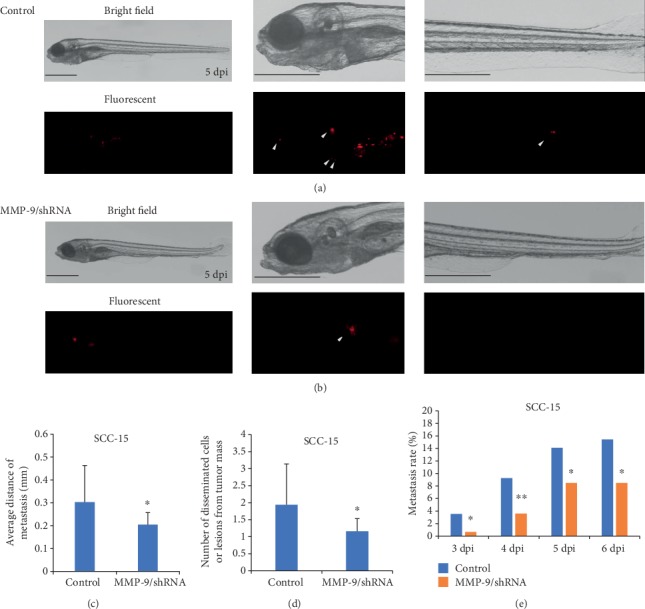
Knockdown of MMP-9 reduced the metastatic distance and number of SCC-15 cells and decreased the metastasis rate in the zebrafish xenograft model. (a, b) The bright-field and fluorescent pictures of whole-body, head, distal tail regions of zebrafish injected with SCC-15/LVRU6MP (*n* = 230; magnification, ×32, ×63) and SCC-15/MMP-9/shRNA cells at 5 dpi (*n* = 290; magnification, ×32, ×63). (c, e) Knockdown of MMP-9 decreased the invasive average distance and number of disseminated cells or lesions at 5 dpi and inhibited the metastasis rate from 3 dpi to 6 dpi. ^∗^*P* < 0.05 and ^∗∗^*P* < 0.01 as compared with the control. Bar = 0.5 mm.

To verify these results again, similar experiments were performed with SCC-15/LVRU6MP and SCC-15/MMP-9/shRNA cells. At 5 dpi, we found that SCC-15/LVRU6MP cells had invaded the eye, pericardium, pronephric region, and distal tail region (Figure 6(a)). However, SCC-15/MMP-9/shRNA cells were detected only in the pronephric region, and no cell invasion was detected in the distal tail region (Figure 6(b)). Quantification analysis revealed that the knockdown of MMP-9 decreased the average metastatic distance and number of metastatic tumor cells or lesions at 5 dpi. Meanwhile, decreasing metastasis rate from 3 dpi to 6 dpi in zebrafish was detected (Figures 6(c)–6(e)). Taken together, the knockdown of MMP-9 in OSCC cells may attenuate the invasive and metastasis ability of OSCC cells *in vivo*. These results in the zebrafish xenograft model are consistent with the behavior *in vitro*.

## 4. Discussion

Cancer cell metastasis has complicated steps, and the detected metastatic tumor mass implies the final step of metastasis in the clinical setting [21]. OSCC is one of most common cancers in humans [31], and its initial lymph node metastasis is common and difficult to be observed at the early stages, possibly due to opaque tissues in human and mice. Importantly, the processes of tumor growth and metastasis in a mouse OSCC model were generally studied after mice are sacrificed. Furthermore, the nude mouse OSCC model has limited utility for large screening of anticancer agents due to many limitations including slow solid tumor growth, high breeding cost, large housing space, and much time for examining mice. However, the early translucent zebrafish embryo develops fast, allows live image *in vivo*, and absorbs compounds in water, and it has been reported that xenografts are not rejected owing to the immature immune system [32]. Therefore, the zebrafish embryo can be used as an ideal tool to observe tumor proliferation, angiogenesis, and metastasis in a real-time manner. In short, after implanting cancer cells into the yolk, the tumor progression includes proliferating, evading attack of immune systems, promoting angiogenesis, intravasating into the circulation, migrating through vasculature, extravasating into the zebrafish body (frequently the head and distal tail region), and proliferating at the new location [33]. From the perspective of tumor metastasis, suppressing any of these steps might prevent cancer cell metastasis. In our study, we established here a novel human OSCC model in zebrafish to investigate the role of the knockdown of MMP-9 in OSCC cells. Additionally, this model may play an important role in evaluating real-time therapeutic effect and high-throughput screening of antioral cancer agents in the future.

In previous studies, MMP-9 has been reported to destroy collagen IV, the main ingredient of basement membranes, and participate in remodeling, angiogenesis, and cancer cell metastasis in some solid tumors [34]. In brief, MMP-9 may act as a tumor promoter in many solid tumors [10–13]. In our study, we found similar results. The knockdown of MMP-9 may effectively inhibit OSCC cell metastasis and invasion in xenografted zebrafish. These evidences confirmed that the role of MMP-9 in OSCC cells is consistent with that in series of cancers and relevant clinical researches [15–18].

As previously described, angiogenesis is an important step of cancer metastasis and facilitates distant metastasis. Studies have shown that tumor-induced angiogenesis was detected after human melanoma cells were injected into the zebrafish embryo yolk and hindbrain ventricle [35]. Moreover, the formation of angiogenic sprouts and neovascularization could be observed by implanting mouse melanoma cells or colon cancer cells into the embryo perivitelline space. And this process was suppressed by SU5416 (vascular endothelial growth factor receptor 2 (VEGFR2) inhibitor) [36]. In other studies, Ewing sarcoma cells have also been reported to induce sprouting of the subintestinal vein (SIV) and growth in the zebrafish xenograft model [37]. Thus, we hypothesize that human OSCC cells might invade into the fish body by stimulating sprouting of the SIV. However, the underlying mechanisms that reduction of MMP-9 expression in OSCC cells may inhibit this process remains unclear in the zebrafish model and needs to be further studied.

## 5. Conclusions

In summary, the knockdown of MMP-9 in OSCC cells might inhibit tumor cell metastasis and invasion and reduce the metastasis rate of xenografted zebrafish. It was confirmed that MMP-9 may play a key role in OSCC cell metastasis and invasion *in vivo* and *in vitro*. These results imply that the MMP-9 gene may act as a promising therapeutic target in OSCCs. But underlying molecular mechanisms need to be further investigated in the future study.

## Figures and Tables

**Table 1:** The details of implantation.

Cell line	Fish line	Implantation density (/ml)	Implantation period (hpf)	Number of injected cells	Number of xenografted zebrafish	Implantation site	Temperature (°C)
CAL-27/LVRU6MP	WT	4.0 × 10^7^	48	500-600	120	Yolk sac	29
CAL-27/MMP-9/shRNA	WT	4.0 × 10^7^	48	500-600	120	Yolk sac	29
SCC-15/LVRU6MP	WT	4.0 × 10^7^	48	500-600	230	Yolk sac	29
SCC-15/MMP-9/shRNA	WT	4.0 × 10^7^	48	500-600	290	Yolk sac	29

## Data Availability

The data used to support the findings of this study are included within the article.

## Abbreviations

MMP-9: Matrix metallopeptidase 9
MMPs: Matrix metalloproteinases
OSCC: Oral squamous cell carcinoma
ECM: Extracellular matrix
EGFR: Epidermal growth factor receptor
FBS: Fetal bovine serum
DMSO: Dimethyl sulfoxide
OD: Optical density
BSA: Bovine serum albumin
PTU: Phenylthiourea
hpf: Hours postfertilization
dpi: Days postinjection
SIV: Subintestinal vein
VEGFR2: Vascular endothelial growth factor receptor 2
DAB: Diaminobenzidine.
WT: Wild-type.
TU: Tuebingen.

## Supplementary Materials

Supplementary Materials Supplemental Figure 1: the shRNA expression clone datasheet. (a) The vector information. (b) The shRNA target sequences. Sequence 33 was selected for subsequent lentivirus packaging and transfection. Supplemental Figure 2: knockdown of MMP-9 in CAL-27 cells could slightly inhibit the metastasis rate from 3 dpi to 5 dpi, but the results were not statistically significant. ns: no significance.
