# Supplementary material for: Knockdown of Matrix Metallopeptidase 9 Inhibits Metastasis of Oral Squamous Cell Carcinoma Cells in a Zebrafish Xenograft Model
Source: Biomed Res Int. 2020 Apr 15;2020:4350783. doi: 10.1155/2020/4350783 (PMC7183015; doi:10.1155/2020/4350783)
Supplement: Supplementary Materials — Supplemental Figure 1: the shRNA expression clone datasheet. (a) The vector information. (b) The shRNA target sequences. Sequence 33 was selected for subsequent lentivirus packaging and transfection. Supplemental Figure 2: knockdown of MMP-9 in CAL-27 cells could slightly inhibit the metastasis rate from 3 dpi to 5 dpi, but the results were not statistically significant. ns: no significance. [file 4350783.f1.pdf]

Supplemental Materials

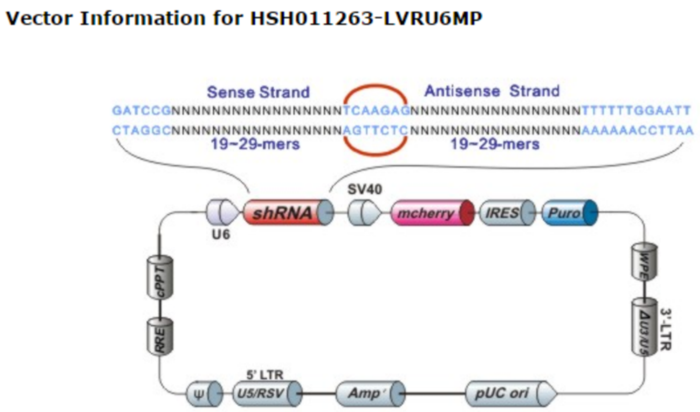

(a)

shRNA Target Sequences

Clone Name	Symbol	Location	Length	Target Sequence
HSH011263-5-LVRU6MP(OS398288)	MMP9	199	21	gcgtggagagtcgaaatctct
HSH011263-6-LVRU6MP(OS398289)	MMP9	1082	21	ggtaaggagtactcgacctgt
HSH011263-7-LVRU6MP(OS398290)	MMP9	1242	21	gcttagatcattcctcagtgc
HSH011263-8-LVRU6MP(OS398291)	MMP9	1312	21	gcataaggacgacgtgaatgg
HSH011263-34-LVRU6MP(OS714976)	MMP9	1139	21	gctaccacctcgaactttgac
HSH011263-35-LVRU6MP(OS714977)	MMP9	1270	21	gctcatgtaccctatgtaccg
HSH011263-32-LVRU6MP(OS714974)	MMP9	331	21	gggcagattccaaacctttga
HSH011263-33-LVRU6MP(OS714975)	MMP9	511	21	agacatcgtcatccagtttgg

(b)

Supplemental Figure 1: *The shRNA expression clone datasheet.* (a) The vector information. (b) The shRNA target sequences. The sequence 33 was selected for subsequent lentivirus packaging and transfection.

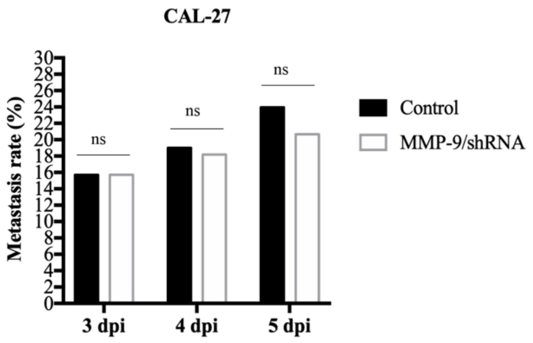

Supplemental Figure 2: *Knockdown of MMP-9 in CAL-27 cells could slight inhibit the metastasis rate from 3 dpi to 5 dpi, but the results were not statistically significant.* ns, no significance.
